# Do non-native species contribute to biodiversity?

**DOI:** 10.1371/journal.pbio.2005568

**Published:** 2018-04-17

**Authors:** Martin A. Schlaepfer

**Affiliations:** Institute of Environmental Sciences, University of Geneva, Geneva, Switzerland

## Abstract

The Convention on Biological Diversity (CBD) emphasises the role of biodiversity in delivering benefits essential for all people and, as a result, seeks to safeguard all life-forms. The indices that are used to measure progress towards international conservation and sustainability goals, however, focus solely on the ‘native’ component of biodiversity. A subset of non-native species can cause undesirable economic, social, or biological effects. But non-native species also contribute to regional biodiversity (species richness and biotic interactions) and ecosystem services. In some regions and cities, non-native species make up more than half of all species. Currently, the contributions of these species to biodiversity and ecosystem services are overlooked. Here, I argue that biodiversity and sustainability indices should include all species. This is not only consistent with definitions of biodiversity but also will promote the idea that long-term, sustainable, human well-being is intricately tied to benefits derived from nature.

Humans have a long history of protecting certain elements of nature. The concepts and values underlying conservation initiatives, however, have changed repeatedly [1]. Conservation efforts in the 20th century focused primarily on preserving landscapes free of human influence and on preventing the erosion of biodiversity, with an emphasis on protecting rare species from extinction. The last 20 years have seen the emergence of additional concepts that emphasise the resilience of nature and the ‘services’ that nature contributes to human well-being [2,3]. These novel approaches are promoted by some conservation leaders under the assumption that they will broaden the social support for conservation goals [4,5]. The advent of more socially inclusive approaches to conservation biology raises interesting and profound questions regarding the dimensions of the living world we seek to preserve and the political process that is used to specify appropriate conservation objectives.

Non-native species, and how they are valued, are at the heart of these ongoing debates. For the last several decades, non-native species have been portrayed by scientists primarily as a threat to society because a subset can cause economic harm, human-health issues, or the loss of native biodiversity [6,7]. The view that non-native species are potentially undesirable persists in indicators used to track progress towards targets of the Convention on Biological Diversity (CBD), where they only appear as a numerical predictor for future invasion events (Aichi Target 9) [8].

More recently, scientists have also documented the potential positive contributions of non-native species to regional species richness [9,10], conservation goals [11], and to the ecosystem services they contribute to certain stakeholders within society [12–14]. In some instances, non-native species are rapidly appreciated for their cultural ecosystem services. For example, citizen groups have lobbied for the protection of non-native Eucalyptus trees in California and non-native dingos in Australia [11]. The potentially positive contributions of non-native species to biodiversity and to the long-term welfare of humans are missing from current biodiversity indicators (Table 1). This raises the following questions: Are non-native species part of ‘nature’ or ‘biodiversity’ that we wish to preserve? If so, can they be integrated into a conservation planning process in a way that recognises their potential for undesirable effects but also captures their potential positive contributions to biodiversity and society?

**Table 1:** A synthesis of the role of non-native species in biodiversity indicators and assessments related to species richness at global and regional scales.

Name of Indicator or Study	Ref.	Use	NNS	Comment
**Global**				
Living Planet Index	[15,16]	To inform Aichi Biodiversity Target 12	Included	Non-native populations make up 1.5% of tracked populations (286/18,427; July 2017 email from Stefanie Deinet to me, unreferenced citation. See Acknowledgments).
IUCN Global Red List	[17]	To identify globally threatened species	Excluded	The IUCN protocol does not normally consider populations outside of a species’ native range in the evaluations of a species’ extinction risk. Consequently, the extinction risk of species with significant non-native populations [18] will be overestimated. Furthermore, when the global IUCN data set is disaggregated regionally [19], it does not include non-native species in each region and thus likely results in an overestimate of the percentages of all species (native + non-native) that are threatened.
BII	[20]	To inform Planetary Limit of biodiversity	Excluded	BII value increased by 10% when ‘novel’ species are assumed to be functionally equivalent to ‘native’ species [21].
Global Study on State of Biodiversity	[22]	To project future biodiversity, by biome	Excluded	Defines biodiversity as ‘all terrestrial and freshwater organisms’, yet excludes NNS.
Wild Bird Index	[23,24]	To inform Aichi Biodiversity Target 12	Excluded	Listed on the Biodiversity Indicators Partnership site as an indicator applicable for national use and included in CBD indicators (https://www.bipindicators.net/indicators/wild-bird-index, Accessed 4 April 2018).
City Biodiversity Index (Singapore)	[25]	To measure biodiversity in cities, under CBD	Excluded	Five indicators focus on species richness of different taxa. All focus exclusively on native species and no rationale is provided for excluding NNS.
**Regional**				
EU Common Birds Indicator	[26]	To measure health of environment, sustainability and to inform effectiveness of European Union Directives and Common Agricultural Policy	Excluded	Rationale for excluding NNS: ‘Non-native species are excluded, being an unnatural component that doesn´t contribute to the quality of the avifauna’. (http://www.ebcc.info/index.php?ID=491, Accessed 13 March 2018).
IUCN Regional and National Red Lists	[27]	To identify regionally threatened species	Excluded	NNS are assigned the *Not Applicable (NA)* code and therefore are not eligible for evaluation. This likely leads to a regional overestimate of the percentages of total species threatened.

**Abbreviations:** BII, Biodiversity Intactness Index; CBD, Convention on Biological Diversity; IUCN, International Union for Conservation of Nature; NNS, non-native species.

Biodiversity is a complex notion and the indicators that are used to track it [16] are a reflection of available information, objectives, and values, which all can vary culturally and with time [3,28]. However, it is important to ask under what circumstances it might be scientifically and politically desirable to modify some biodiversity indicators to include all species in light of ongoing preparations for the Post-2020 Strategic Plan of CBD, ongoing assessments by the Intergovernmental Science-Policy Platform on Biodiversity and Ecosystem Services (IPBES), and forthcoming national implementation of the Sustainable Development Goals (SDGs).

## Non-native species as an integral component of biodiversity

There are several reasons why non-native species should be considered part of biodiversity and included in biodiversity and sustainability indices.

First, the absence of non-native species from biodiversity indices stands in contradiction to the CBD and SDGs. The CBD definition of biodiversity (Article 2) encompasses the biological dimensions of the world (genes, species, ecosystems and their interactions), but it makes no distinction between native and non-native life forms, nor does it refer to notions of ‘intactness’, which forms the basis for excluding non-native species from the Biodiversity Intactness Index (Table 1). Aichi Target 2 and Goal 15.9 of the SDGs require national and local governments to account for ‘the diverse values of biodiversity’.

Second, non-native species should be included in key biodiversity indices because they represent large fractions of modern ecosystems and regional species-pools. Non-native plants and birds can make up 50% or more of species in some urban [29,30], insular [9,31,32], and old-field [33] environments. There is a risk that regional policy makers in areas in which non-native species comprise a significant component of the landscape will perceive biodiversity indices to be irrelevant if they are based solely on native species.

**Fig 1:**
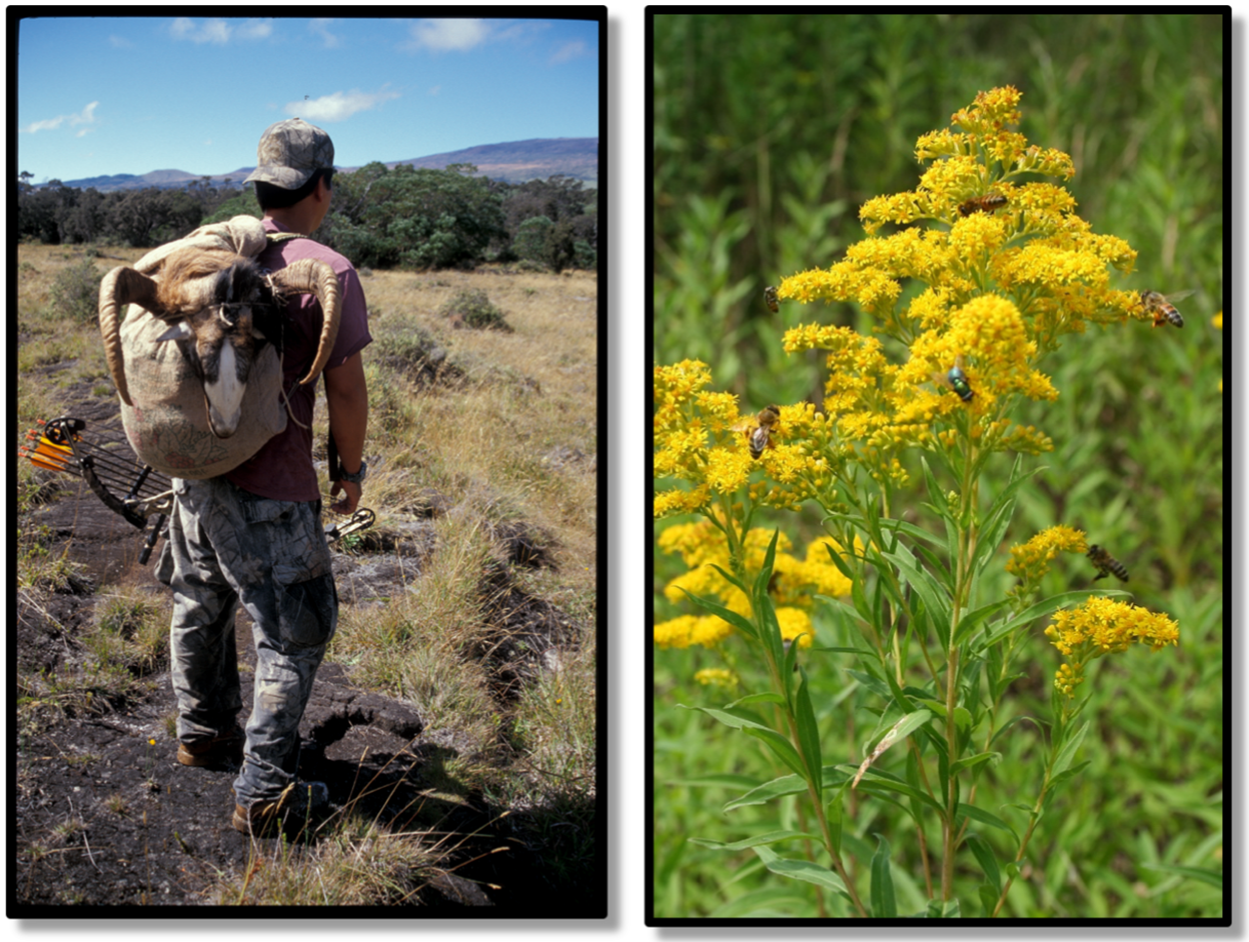
Left: A bow hunter on the Big Island of Hawaii with his catch, a non-native mouflon (feral sheep, *Aries* sp.). Mouflon threaten native plant species that have not evolved to resist mammalian herbivory. Right: Introduced goldenrod (*Solidago gigantea*) population near the city of Geneva, Switzerland. Goldenrod displaces native plants on a local scale and thus is considered invasive in Switzerland. But it is also appreciated for its ornamental and medicinal properties, and it serves as a resource for insects (hymenoptera and diptera, as seen in photograph). Current biodiversity indices and assessments capture the negative aspects of such non-native species (i.e., their potential for harm) but not their contributions to biodiversity (increase in regional species richness, interaction with other species) nor the ecosystem services (provisioning, regulating, cultural, supporting) that are socially and biologically relevant. Photos by author.

Finally, and perhaps most importantly, society’s motivations for the conservation of biodiversity are evolving and the indicators used to measure the state of the environment and progress towards our goals should too. Biodiversity indices will need to encompass all species if they are to remain socially relevant and illustrate the full gamut of what are now called ecosystem services (and disservices), or nature’s contributions to people [3] (Fig 1).

## Drawbacks of accounting for non-native species

Some will argue that if non-native species are considered as an integral part of biodiversity then, by extension, it will be harder to make the case that non-native species are potentially problematic. Such a change might also be viewed as devaluing conservation efforts that focus on native species. Others have argued that some fraction of the negative impacts of non-native species may be underestimated and will only be known with time [7]. These are legitimate concerns.

Accounting for non-native species does not imply that they are inherently desirable, nor that native and non-native species are biologically or culturally interchangeable. Nor does counting all species as an initial step to describing the environment preclude scientists from conducting risk assessments of different species or groups of species regarding undesirable outcomes [7]. But to exclude non-native species a priori and without justification from indices (Table 1) and assessments of the state of the environment, as is sometimes done [22,34], prevents any subsequent debate about their relative merits.

## Moving forward

There are several steps that can be taken to better align future conservation planning with the mission of CBD and the SDGs.

- Data used for biodiversity assessments and conservation planning should, initially, include all species. This will not only ensure that indices are representative of the environments they seek to characterise, but it will also align species richness and identity information with the functional information that is increasingly being measured remotely [35].
- Researchers should continue to investigate the extent to which non-native species influence the flow of ecosystem services and contribute to human well-being. One overlooked area of research is how including non-native species may alter existing biodiversity indices. Including non-native species into the Biodiversity Intactness Index can lead to a 10% improvement in the index score [21], and consideration of non-native populations in International Union for Conservation of Nature (IUCN) protocols will reduce a species’ global risk of extinction [18]. These examples hint that some existing index scores may be overly negative.
- Panels that decide which indicators to use when assessing progress towards global conservation goals, especially those that establish a link with human well-being (e.g., Aichi Targets), should be composed of specialists and laypersons from a range of backgrounds. Some indicators should reflect the preference of certain stakeholders for notions of ‘nativeness’ or ‘pristineness’, while others should capture all species found in modern ecosystems and their contributions to human well-being.
- Biodiversity and sustainability assessments that exclude non-native species [22,36,37,38] should systematically specify that they are tracking ‘native biodiversity’ and not ‘biodiversity’, as these terms are not synonymous.

The idea of considering non-native species in biodiversity assessments remains controversial [39] in part because it runs against decades of studies alerting policy makers and the public to the potential dangers associated with these species. Scientifically and politically, however, it may be the best thing to do, as it will ultimately ensure that indices and the databases that underlie them cover all dimensions of the living world and remain relevant to groups of stakeholders that extend beyond conservation biologists. The discussion about how to best integrate non-native species into biodiversity indicators will need to take place not only within a scientific debate about the contributions of these species to society but also a broader social debate about what type of nature we need to ensure a good life for present and future generations.

## Abbreviations

CBD: Convention on Biological Diversity
IPBES: Intergovernmental Science-Policy Platform on Biodiversity and Ecosystem Services
IUCN: International Union for Conservation of Nature
SDG: Sustainable Development Goal
